# Quick and safe: why a k-wire-extension-block-fixation of a bony mallet finger is the favoured treatment

**DOI:** 10.1007/s00402-023-05119-y

**Published:** 2023-12-26

**Authors:** Maximilian C. Stumpfe, Nadine Suffa, Pauline Merkel, Ingo Ludolph, Andreas Arkudas, Raymund E. Horch

**Affiliations:** Department of Plastic and Hand Surgery and Laboratory for Tissue Engineering and Regenerative Medicine, University Hospital Erlangen, Friedrich Alexander University Erlangen-Nürnberg FAU, Krankenhausstrasse 12, 91054 Erlangen, Germany

**Keywords:** Mallet finger, Intervention, Kirschner-wire-fixation, Bony extensor tendon rupture, Mallet fracture

## Abstract

**Introduction:**

Mallet fingers are the most common tendon injuries of the hand. Bony avulsion distal finger extensor tendon ruptures causing a mallet finger require special attention and management. In this monocentral study, we analyzed the clinical and individual outcomes succeeding minimal invasive k-wire extension block treatment of bony mallet fingers.

**Materials and methods:**

In a retrospective study, we sent a self-designed template and a QUICK-DASH score questionnaire to all patients, who were treated because of a bony mallet finger between 2009 and 2022 and fulfilled the inclusion criteria. A total of 244 requests were sent out. 72 (29.5%) patients participated in the study. Forty-five men and twenty-seven women were included.

**Results:**

98.7% (*n* = 75) of the cases were successfully treated. Patients were highly satisfied with the treatment (median 8.0; SD ± 2.9; range 1.0–10.0). Based on the QUICK-DASH score, all patients showed no difficulties in daily life. The extent of avulsion did not influence the outcome.

**Conclusion:**

We conclude that the minimally invasive treatment of a bony mallet finger should be offered to every patient, because it is safe, fast, and reliable. Thus, we propose to perform extension-block pinning independently of the articular area.

## Introduction

A mallet finger or a drop finger results from a sudden flexion or hyperextension force on an extended distal phalanx with or without a bony avulsion of the distal tendon insertion [1–3]**.**

**Fig. 1:**
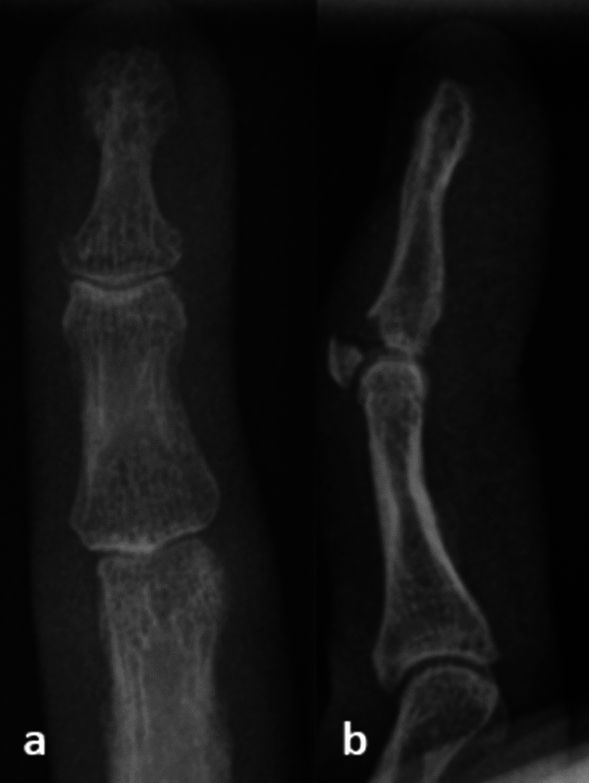
X-ray of a bony mallet finger: a posterior–anterior and b lateral

Extensor tendon disruption of the fingers in zone 1 according to Verdan occurs as a closed injury in 75% of the cases and is the most common tendon injury of the hand [4–6]. A bony mallet finger is the result of an avulsion of the extensor tendon from the distal finger phalanx with a small or large fragment of bone attached to the avulsed tendon (Fig. 1) and needs special attention and treatment.

**Fig. 2:**
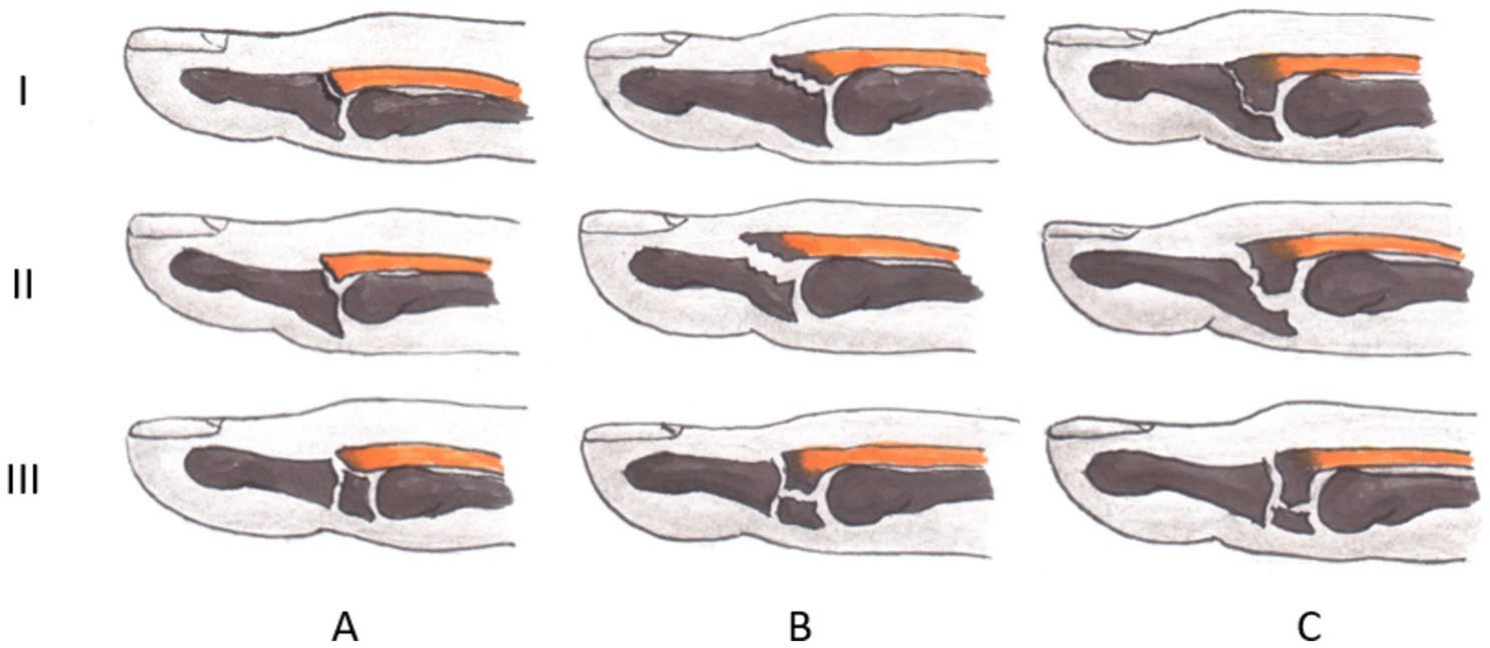
Representation according to Wehbé and Schneider of different bony mallet fingers based on the joint surface involved (subtype A: < 1/3; subtype B: 1/3–2/3; subtype C: > 2/3) and the presence of a subluxation (type 1: no volar subluxation; type 2: volar subluxation) and growth plate fracture (type 3) ((modified from: S. Salazar Botero et al., Review of Acute Traumatic Closed Mallet Finger Injuries in Adults [7])

The extent of the bony distal interphalangeal (DIP) joint avulsion can vary from a small bony fragment up to displaced juxta-epiphyseal fractures of the distal phalanx with an overlying nail bed laceration (so-called Seymour frature), thus determining the different treatment forms according to the mentioned classifications (Fig. 2).

Although in the current clinical practice, many mallet finger injuries are managed non-surgically with various splints, full recovery of the extension capacity is not achieved as long as the oblique retinacular ligament and the lumbrical tendon merging into this ligament are not relaxed. If left untreated, mallet fingers lead to a swan neck deformity. Similar to other entities, secondary finger joint stiffness—in partially non-physiological—and dysbalances are difficult to correct [8–11]. Nevertheless, to avoid the sequelae of swan neck deformities and to optimize treatment results, minimally invasive treatment is recommended for either an acute or a chronic mallet finger or as a salvage procedure after of failed prior treatment [12].

Distal extensor tendon disruption causes the disability to actively extend the DIP joint, and therefore, the distal phalanx remains in a flexed position [5]. While the pathogenesis is clearly understood, there is no consensus regarding the optimal therapy. As a result, there is a debate between conservative therapy with immobilization by solely splinting and minimally invasive interventions [13]. In addition, open procedures with using a plate have been described [14]. Special circumstances such as the incision pattern as well as the consequences of tourniquet ischemia and suture materials must be considered [15–18]. Ultimately, the aim of the therapy is to regain the ability of complete DIP extension while preserving flexion in the DIP joint preventing secondary swan neck deformities at the proximal interphalangeal (PIP) joint [5]. In this context, the measured extension of the DIP joint varies among different authors to define what can be considered a successful treatment. For example, an extension deficit smaller than 20° but also smaller than 15° or 10° is set as the upper limit value for extension insufficiency [2, 19–21]. Therapeutic success depends largely on patient compliance when conservative therapy is attempted, but is hampered by insufficient relaxation of the lateral tractus in a Stack device [20, 21].

Conservative treatment consists of the immobilization of the DIP joint using a splint for 8–12 weeks [20–22]. The patient must be informed that the DIP joint must continuously remain in a slight hyperextension position—during daily skin care and during medical checks [20, 21]. If insufficient immobilization of the DIP joint occurs because of a missing hyperextension or because of the smallest movement in the splint, scar tissue forms in the fracture gap and the fracture heals in excess length. This can lead to a further loss of function [22, 23]. Surgical therapy is indicated when the affected fractured joint area is ≥ 30% [24].

This study evaluates the results of minimally invasive treatment of mallet fractures by extension block pinning according to Ishiguro (also known as “door stop osteosynthesis”), size-independent in regard to the affected joint surface.

## Materials and methods

### Collective

We retrospectively enrolled all patients in this study who received an extension-block technique for bony mallet fingers between January 2009 and July 2022. We treated a total number of 267 patients with a mallet fracture. Exclusion criteria was a follow-up period less than 6 months, wherefore 23 patients were excluded from the study. The remaining patients were contacted and asked to return a specially prepared questionnaire and a goniometer including a patient instruction to assess the range of motion of the affected finger. Of 244 sent questionnaires and templates, 72 (29.5%) were returned. All acute and delayed injuries (> 2 weeks) were in this study.

### Operating technique and follow-up treatment

**Fig. 3:**
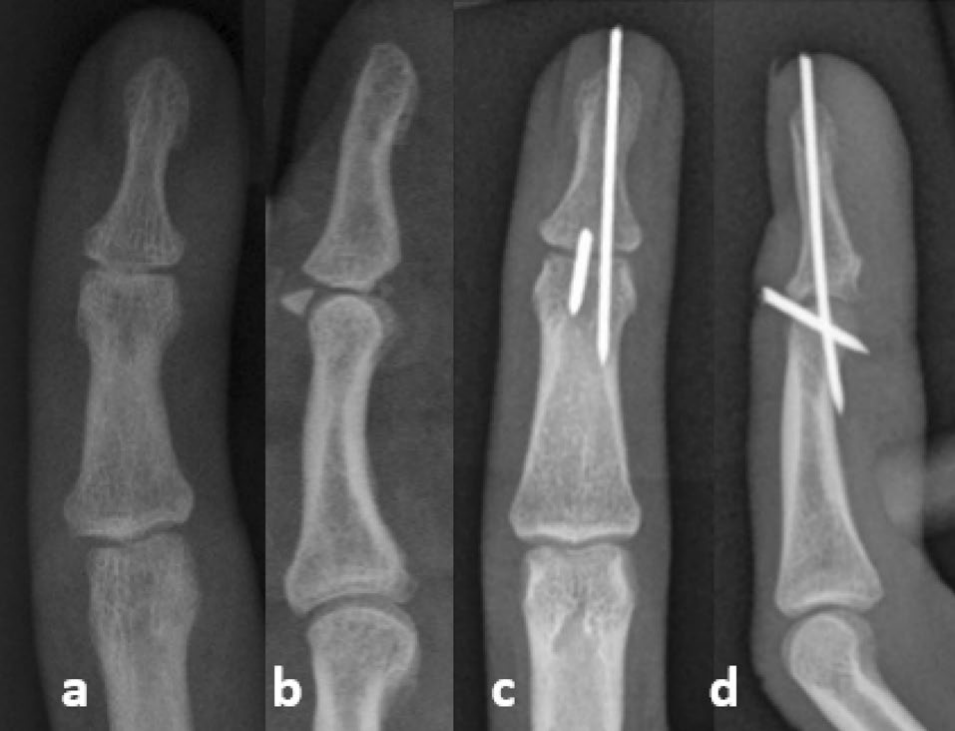
Exemplary illustration of a pre-op X-ray (**a** posterior–anterior; **b** lateral) and post-op X-ray control (**c** posterior–anterior; **d** lateral)

K-wire transfixation according to Ishiguro was performed under local anesthesia [25, 26]. For this purpose, a K-wire (strength 1.0 mm) is first inserted into the head of the middle phalanx in a flexed position of the DIP joint. Afterwards, an extension of the DIP joint was performed; a slight hyperextension can support the attachment of the bony fragment to the base of the phalanx. The already inserted wire serves as a counter bearing for the bony fragment. Then, the temporary transfixation is performed by drilling in the second K-wire, transfixing the distal interphalangeal joint (Fig. 3). The K-wires were then shortened below skin level.

After 6 weeks, the inserted osteosynthesis material must be removed. After the removal of the wires, no further splint immobilization was necessary and a physiotherapeutic therapy was recommended.

### Functionality survey

**Fig. 4:**
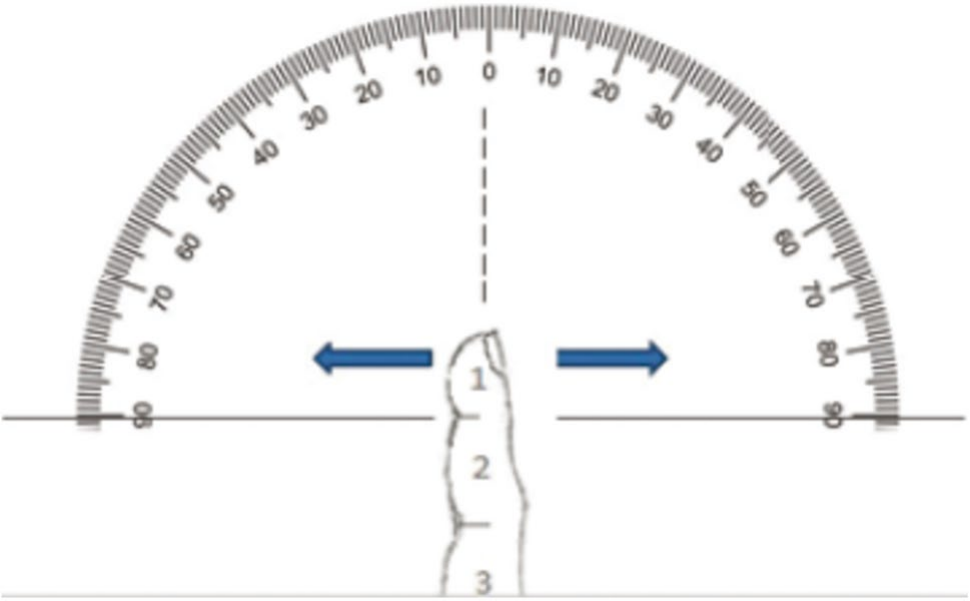
Self-designed goniometer to determine the range of motion through the participants [5]

The self-designed questionnaire and a goniometer (Fig. 4) including an instruction form to determine the range of motion of the distal joint were sent to the participants. The simple procedure for self-evaluation of extension and flexion in the distal joint was explained step by step to the patients.

All participants listed the degrees of motion in a separate sheet. The QUICK-DASH score including work module was used to measure daily functional limitation. Subjective satisfaction with treatment outcome and the presence of pain were separately assessed using a scale of 0 (unsatisfied/no pain)–10 (very satisfied/ worst pain). The outcome of the DIP joint extension was classified into four groups (excellent, good, satisfactory, and insufficient) using the Crawford classification [27]. Complications could be submitted by patients as free text. For example, wound-healing disorders, infections, fever, bleeding, or allergies were mentioned.

### Statistical analysis

Descriptive statistics were expressed as median, standard deviation and range. D'Agostino–Pearson normality test was performed. An unpaired t test was used, as appropriate. Significance was given by a *p* value < 0.05. Statistical analysis was conducted with GraphPad Prism 9.0 (GraphPad Software Inc., La Jolla, CA, USA).

## Results

**Fig. 5:**
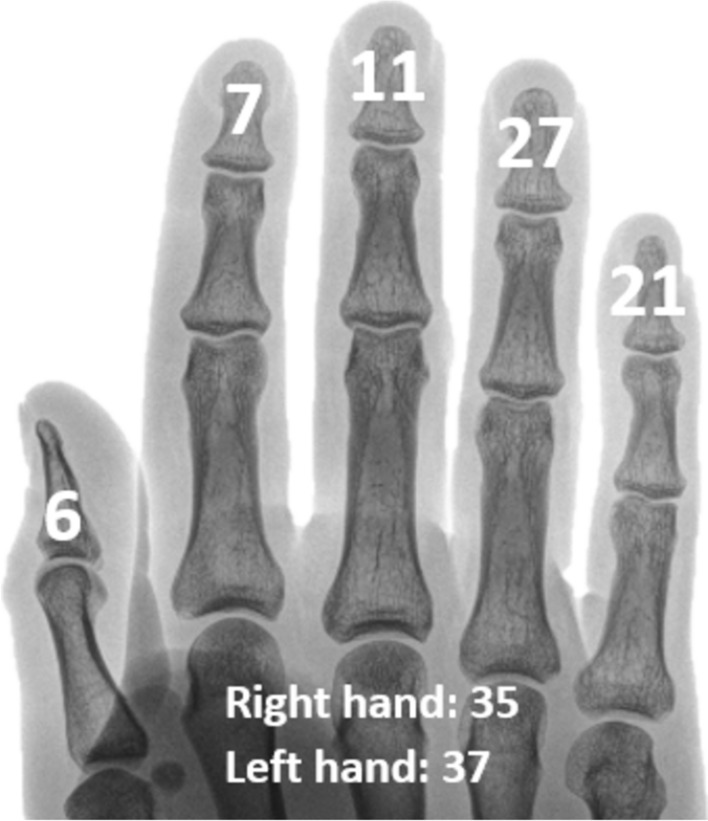
Overview of the frequency distribution of the affected fingers and hand

The average time between minimally invasive treatment of the mallet finger and receipt of the questionnaire was 47 months (± 33.9; range 3–122 months). The patients in the study were on average 44 years old (range 18–85 years). Forty-five men (62.5%) and 27 (37.5%) women participated in the study. The affected fingers are illustraded in Fig. 5. Three patients had several fingers affected.

An acute treatment (less than 2 weeks) was seen in 84.6% of the cases (mean 5.6 days; range 1–13 days). The remaining mallet fingers (15.4%) were treated more than 2 weeks (mean 18.5 days; range 14–32 days) after the accident.

We did not observe side effects or major complications which would have needed additional treatment. Radiologically, all avulsion fractures were healed at the time of K-wire removal.

Given the results of our survey, on average, our patients were highly satisfied with the treatment (median 8.0; range 1–10). Study participants reported no pain (median 0; range 0–6). Based on the QUICK-DASH score, all patients showed no difficulties in daily life. The median QUICK-DASH score was 11.0 points (range 11–30). Fifty-eight participants (80.5%) were employed at the time of the survey and scored 4.0 points (range 4–12) on the additional module of the QUICK-DASH score for employees. According to the Crawford classification, 64 patients (84.3%) obtained an excellent outcome from the treatment. Nine cases (11.8%) achieved a good result. Satisfactory patient outcomes were achieved in 2 cases (2.6%). One treatment (1.3%) was unsuccessful because of a remaining deficit of extension.

The evaluation of complications showed no postoperative problems in 64 cases (84.2%). In 15.8% (*n* = 12) of the patients, grade 1 complications according to the Clavien–Dindo classification were observed. Two cases (16.7%) showed post-interventional infections, wound-healing disorders were reported by four patients (33.3%), and six cases (50.0%) reported hypesthesia in the area of the distal phalanx, without the need for early K-wire removal.

The evaluation of the affected articular surface area can be seen in Table 1. On an average 41% of the entire joint surface was affected. The range was from 25 to 68%.

**Table 1:** Evaluation of the affected articular surface area from bony mallet fingers

	Joint size (mm)	Fragment size (mm)	Percentage (%)
Minimum	3.6	1.3	25
Median	6.0	2.5	41
Maximum	11.6	4.6	68
Std. Deviation	1.4	0.7	0.08

Due to the above-mentioned affected articular surface (Table 1) and the present subluxation of the bony fragment (according to type II by Wehbé and Schneider) in 100% of the cases, the following classification results was according to Wehbé and Schneider: Subtype A type II in 3%, subtype B type II in 95.5% and subtype C type II in 1.5% of the cases.

## Discussion

For treating mallet fingers—whether with or without a bony component—both anatomical and patient-specific aspects must be considered in the therapeutic procedure. It is generally agreed within the current literature that bony mallet fingers should be managed surgically when more than 30% of the articular joint surface is affected and joint subluxation can be detected [28]. This is also true for acute open mallet fractures or after a failed non-invasive treatment via  of splinting. It is also discussed to treat patients conservatively, even if more than 30% of the joint surface are affected [29, 30]. However, Thillemann et al. themselves point out at the end of their study that splinting does not adequately prevent secondary subluxation of the joint [29]. For this reason, the authors of the present work consider conservative treatment of mallet fingers with more than 30% joint surface involvement to be contraindicated. Consistent immobilization of the affected distal interphalangeal joint is obligatory for the success of the therapy [31]. From the perspective of the authors, immobilization in a splint cannot provide the required stability. Inadequate therapy may lead to a swan neck deformity caused by an imbalance in the distribution of the extensor force between the PIP and DIP joints. The compliance of patients is crucial for the success of conservative treatment [21]. However, despite high patient compliance, there is a risk for a dislocation or movement of the splint, resulting in a lack of hyperextension in the distal interphalangeal joint. The results of an insufficient conservative therapy are difficult to treat. Therefore, minimally invasive treatment for secured immobilization seems to be indicated in most cases. Temporary transfixation of the DIP joint may induce transient stiffness, but resolves with active movement over time. In addition to the above aspect, a temporary minimally invasive treatment can counteract the traction of the intrinsic muscles on the distal interphalangeal joint and thus on the bony fragment [22, 32]. This leads to prevention of pseudarthrosis and consequently resulting in an elongation of the extensor tendon with an associated loss of function of the distal interphalangeal joint with necessary subsequent operations [22, 33]. The low rate of complications and the absence of major complications underline the value of this treatmenat option. According to the classification of Greyman et al., the authors consider an extension deficit smaller than 20 degrees successful. Agreeing to Greyman et al., 98.7% (*n* = 75) of our cases were successfully treated.

The reliability of self-measurements by patients is already discussed in the literature and can be seen as an appropriate and easy tool. Richards et al. tested 178 students to determine whether a self-measurement is reliable compared to a measurement by someone else. They showed a high degree of reliability and repeatability as well as a high correlation with the measurements made by someone else [34]. In a literature review Lu et al. also stated that there is equal diagnostic accuracy when the patient performs the examination [35].

The retrospective study design and the small number of cases despite a large case number in our clinic are limitations of this study. A clinical examination of the distal interphalangeal joint by the patients themselves is a possible source of error, but as a potential method of data collection, it is reasonable and easy to perform. Future studies could include a control group of conservatively treated lesions.

Based on the results of the patient survey in addition to our clinical observations, we conclude that the minimally invasive treatment of a bony mallet finger with k-wire extension blocks (door stop osteosynthesis) should be offered to every patient with a bony avulsion extensor tendon injury, because it is safe, fast, and reliable. According to the presented results of different avulsion fracture types, we propose to perform extension block pinning independent of the afflicted articular area.

## Data Availability

The data presented in this study are available upon request from the corresponding author. The data are not publicly available due to the number of records.
